# The Treatment of Gastric Ulcer

**Published:** 1893-01-14

**Authors:** 


### ROYAL SOUTHERN HOSPITAL, LIVERPOOL.

### The Treatment of Gastric Ulcer.

Ulcer of the stomach is of such frequent occurrence that a few remarks on the treatment will perhaps be acceptable, but before entering on a discussion as to the method of treating these cases it will be interesting to notice one or two points. In the first place, the frequency of this complaint in domestic servants. We find at the Southern Hospital the very great majority are of this calling. Now, why should this be the case? It surely cannot be due to insufficiency or unsuitability of food, for our domestics fare far better in this respect than many of the artisan class, and this class is not so affected with gastric troubles No, we think the quality and quantity of the food are out of the question; it is probably due to the underground kitchens and unhealthy surroundings, or to the constant running up and down stairs, or a combination of both these causes. The problem is a difficult one to solve; nevertheless, the fact remains. Another point is the frequency with which these cases suffer from anæmia and chlorosis, and it will be interesting to consider whether we have the anæmia as a primary trouble, with gastric ulcer as a secondary complication, or the reverse. We are certainly inclined to the former view, where we have an impoverished condition of the blood, insufficient to nourish the delicate mucous membrane of the stomach, and thus leading to an unhealthy condition of the same. We have met a number of cases where this condition of anæmia is brought about by eating raw rice, with the idea that to have a delicate transparent skin without colour is the acme of bliss and the perfection of beauty.

Now, with reference to the treatment, we will first of all discuss the dietetic treatment as it is carried out at the hospital. All solid food is absolutely forbidden. The staple diet in these cases is milk, and one pint of this is allowed in the twenty-four hours, diluted with the same quantity of water, or barley water, and this is given in small quantities, and at short intervals during the day—say five ounces every three hours. If the patient should complain of hunger this quantity is increased to a pint and a-half of milk, or even two pints, diluted to the same extent, but with more frequent feedings, very often every two hours. There is a great desire on the part of the patient to have the feedings warm, but this we find is not wise. After a period varying from a fortnight to three weeks, accordingly as the symptoms abate, viz., vomiting, pain, &c., we gradually feel our way with more solid food. In some cases a custard is allowed, and in others a little stale bread and butter. The effect of this increase in diet is watched most carefully. Should any discomfort arise the patients are put back on the milk and water for a few days longer, but this is the exception rather than the rule. Then, if all goes on well, fish, chicken, and such articles of diet are allowed until they are quite able to take the regulation diets allowed by the hospital. In a very few cases we have to resort to rectal feeding, for example, where even the milk and water cause pain and vomiting, and in these cases it is two and a-half to three ounces of pancreatized milk per rectum every two or three hours, combined with nutrient suppositories.

The medicinal treatment we consider quite secondary to the dietetic, but in the acute stage, where we have pain after food, vomiting, &c., great benefit is often found from codein, gr. ½, argent oxidi, gr. ½, made up in the form of a pill, and one three times a day a few minutes before food, and a blister over the epigastrium as a local means of relieving pain. The bowels are often obstinate, and for this castor oil, pulv. glycyrrhizae co., and simple enemas are useful. The anæmia and chlorosis are combated by the various preparations of iron, but these must only be given when the stomach has to a certain extent recovered its tone, and perhaps the most easily assimilated of the iron preparations is the saccharated carbonate, given in the form of powder spread upon bread and butter, or a s a mixture suspended in mucilage.

During this treatment we cannot emphasise too much the importance of absolute rest. This is more than half the battle in grappling with these cases ; by absolute rest we mean confinement to bed, and that for three weeks, if necessary. By this means all tight and constricting clothing is impossible, thus giving the stomach full play, and allowing and giving complete muscular rest. We find that some patients actually get fat and gain in weight on the spare diet of milk and water while kept in bed.

